# The costs of dementia in England

**DOI:** 10.1002/gps.5113

**Published:** 2019-04-24

**Authors:** Raphael Wittenberg, Martin Knapp, Bo Hu, Adelina Comas‐Herrera, Derek King, Amritpal Rehill, Cheng Shi, Sube Banerjee, Anita Patel, Carol Jagger, Andrew Kingston

**Affiliations:** ^1^ Personal Social Services Research Unit (PSSRU), Department of Health Policy London School of Economics and Political Science London UK; ^2^ Centre for Dementia Studies, Brighton and Sussex Medical School University of Sussex Brighton UK; ^3^ Anita Patel Health Economics Consulting Ltd London UK; ^4^ Institute for Ageing Newcastle University Newcastle upon Tyne UK; ^5^ China Social Security Research Centre, School of Labour and Human Resources Renmin University of China Beijing China

**Keywords:** costs, cost of illness, dementia, England, health care, social care, unpaid care

## Abstract

**Objectives:**

This study measures the average per person and annual total costs of dementia in England in 2015.

**Methods/Design:**

Up‐to‐date data for England were drawn from multiple sources to identify prevalence of dementia by severity, patterns of health and social care service utilisation and their unit costs, levels of unpaid care and its economic impacts, and other costs of dementia. These data were used in a refined macrosimulation model to estimate annual per‐person and aggregate costs of dementia.

**Results:**

There are around 690 000 people with dementia in England, of whom 565 000 receive unpaid care or community care or live in a care home. Total annual cost of dementia in England is estimated to be £24.2 billion in 2015, of which 42% (£10.1 billion) is attributable to unpaid care. Social care costs (£10.2 billion) are three times larger than health care costs (£3.8 billion). £6.2 billion of the total social care costs are met by users themselves and their families, with £4.0 billion (39.4%) funded by government. Total annual costs of mild, moderate, and severe dementia are £3.2 billion, £6.9 billion, and £14.1 billion, respectively. Average costs of mild, moderate, and severe dementia are £24 400, £27 450, and £46 050, respectively, per person per year.

**Conclusions:**

Dementia has huge economic impacts on people living with the illness, their carers, and society as a whole. Better support for people with dementia and their carers, as well as fair and efficient financing of social care services, are essential to address the current and future challenges of dementia.

**Key points**

- On the basis of the newly available data and refined modelling, we estimate the total annual cost of dementia in England to be £24.2 billion in 2015, £2 billion higher than the previous estimate for 2013.
- Family and other unpaid carers make substantial contributions to the support of people with dementia. Given rapid population ageing, the already substantial demand for, and costs of, unpaid care is expected to increase enormously in the future, which calls for better support for carers.
- The economic impact of dementia weighs more heavily on the social care than on the health care sector and on people with more severe dementia. Fair and efficient financing and provision of social care services is essential to ensure that high‐quality care is delivered in a timely fashion to people with dementia and their families.

## 1 INTRODUCTION

Around 850 000 people currently live with dementia in the United Kingdom,^1^ of whom 660 000 live in England.^2^ Based on demographic changes alone, UK prevalence could exceed^3^ 2 million by 2051. The projected rise in numbers presents major challenges to families, formal care services, and to public and private budgets. Understanding the economic consequences of such a prevalent condition is essential to engaging the public and encouraging policymakers to invest in appropriate treatment, care and support, and preventative actions and research, particularly to find disease‐modifying as well as symptomatic interventions.

Cost‐of‐illness (COI) studies aim to identify and measure all costs of a disease or condition to estimate its total impact on society in monetary terms.^4^ In the case of dementia, this involves, but is not confined to, estimating total costs of health, social, and unpaid care for all people with dementia. COI studies can raise awareness of the substantial and rising financial impact of dementia and shed light on the adequacy or otherwise of responses to it, thereby acting as a lever for potential reprioritisation of resources.

Studies worldwide have estimated the costs associated with dementia. Some studies focussing on particular population subgroups.^5^, ^6^, ^7^, ^8^, ^9^ Two closely related COI studies have been conducted in the United Kingdom,^3^, ^10^ with total costs of dementia estimated at £26 billion in 2013, 24% higher than previously estimated in 2007 (adjusting for inflation and additional coverage, largely due to an increase in the number of people with dementia). Two‐thirds of the cost arose from unpaid care and payments for privately funded social care borne by people with dementia and their families.

These two UK estimates of the costs of dementia relied on data, now over a decade old, derived from a number of small studies each with criteria that excluded certain groups, such as people with severe dementia. Using up‐to‐date prevalence estimates, service utilisation and unpaid care data from multiple sources, and a more refined modelling approach than previously employed, we report the per‐person and total societal costs of dementia in England in 2015.

## 2 RESEARCH METHODS

### 2.1 Overall estimation approach

We sought to estimate the societal costs of dementia in England for 2015, encompassing costs of health, social, and unpaid care. Our modelling refines approaches previously used,^3^ which in turn built upon related studies by research team members. The availability of data for older people (age 65 and over) and younger people (age 35 to 64) varied; hence, different models were used to estimate costs for these two age groups. Although fewer data are available for younger people with dementia, we have included estimates for younger adults to aid comparability with earlier estimates and to be inclusive of all age groups.

**Figure 1:**
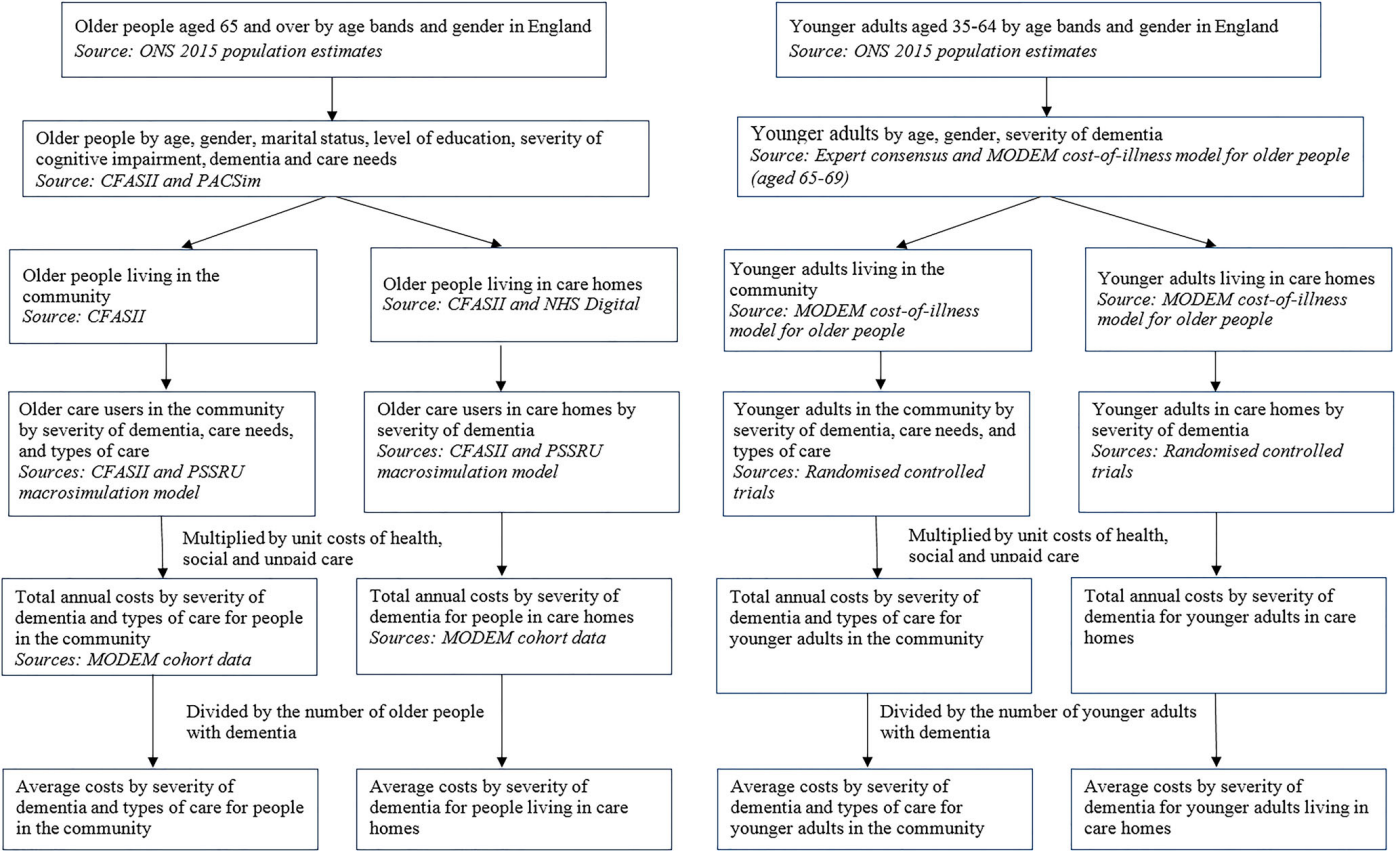
A, Structure of the MODEM cost‐of‐illness model for older people. B, Structure of the MODEM cost‐of‐illness model for younger adults [Colour figure can be viewed at wileyonlinelibrary.com]

Our model for *older people* has three parts. First, we divide the older population into subgroups according to relevant characteristics. Second, we estimate the number of older people with dementia using different types of community care and care home services in each subgroup. Third, we calculate average per‐person cost and aggregate costs for older people with dementia at national level (Figure 1A). We used the best available secondary data sources to derive reliable estimates for (a) the number of older people with dementia in England in 2015, (b) their receipt of health, social, and unpaid care, and (c) costs associated with that care and other related activities. Our model for *younger adults* follows the same approach as for older people but has a simpler structure and provides less detailed cost estimates due to data limitations for this group (Figure 1B).

All three cost categories (health, social, and unpaid care) were estimated separately by severity of dementia (mild, moderate, and severe) and by year since onset of dementia (first year and subsequent years). National Health Service (NHS) costs were split by primary and secondary care. Social care costs were split between publicly and privately funded care. All reported estimates are annual costs for England for 2015, in pounds sterling (£) at 2015 price level. Cost estimates represent a snapshot for 2015, not lifetime costs. Discounting was unnecessary since all costs refer to a 1‐year period.

### 2.2 Data sources

Estimates were derived from multiple sources:

1. Numbers of older people and younger adults in England, disaggregated by age and gender, come from the 2015 population estimates published by the Office for National Statistics.^11^
2. Estimates from the Population Ageing and Care Simulation (PACSim) model^2^, ^12^, ^13^ were used to generate prevalence rates of cognitive impairment by severity and care needs by “interval need” in the older population.^14^ Rates were estimated by age, gender, and education. Full details of validation of the PACSim model have previously been described.^13^
3. The proportion of older people with dementia, by age, gender, and education, and the proportion of those with dementia who receive unpaid care, formal community care, and care home services according to their characteristics (age, gender, education, and severity of cognitive impairment) were estimated using the first wave of the Cognitive Function and Ageing Study (CFASII) data.^15^
4. MODEM cohort data^16^ generated estimates by severity of dementia of weekly and annual costs of health, social, and unpaid care. The cohort comprises 318 people with clinically diagnosed dementia (110 people with mild, 100 with moderate, and 97 with severe dementia) and their main carers, identified from populations served by Sussex Partnership NHS Foundation Trust. Cohort members were interviewed twice, 12 months apart. While some individuals were care home residents, the majority resided in the community. The survey included an adapted version of the Client Service Receipt Inventory (CSRI)^17^ to collect information on service use and support from family and other carers, the revised Bristol Activities of Daily Living Scale (BADLS) questionnaire,^18^ an amended version of the Resource Utilisation in Dementia (RUD) instrument,^19^ and other questionnaires.
5. Service use data from the MODEM cohort were converted to annual costs using figures from the Unit Costs of Health and Social Care.^20^ Data on service use and unit costs are shown in Table S1.
6. NHS Digital data were used on overall numbers of older users of publicly funded home care and care home service users in England. We applied to them the proportion of older home care users and care home residents found by CFASII to have dementia, to ensure estimates are consistent with official figures.^21^
7. For the younger adult model, the prevalence rates of dementia by age and gender and the proportions of people with dementia using different types of services by severity of dementia come from data previously reported.^3^, ^10^

### 2.3 Measurement of dementia

To estimate the numbers of older people with dementia in 2015, we applied prevalence rates by age, gender, and education from the PACSim model, which drew on CFASII data, to ONS population estimates for 2015. We took account of years of education because the prevalence of cognitive impairment varies with years of education. Identification of dementia in CFASII was based on the well‐established AGECAT algorithm.^15^, ^22^, ^23^ Incidence and prevalence rates derived from CFASII relate to this definition. Overall numbers of people with dementia were divided into three severity levels (mild, moderate, and severe) using a breakdown that maps to the conventional Mini‐Mental State Examination (MMSE). As previously,^3^ the following cut‐off points were used: 21 to 26 for mild, 10 to 20 for moderate, and less than 10 for severe dementia. Numbers by severity were further divided by extent of care needs (independent, requiring help less often than daily, requiring help at regular times of the day, and requiring 24‐hour care) based on Isaac and Neville's *interval needs* classification^14^ to ensure greater accuracy in cost calculation.

### 2.4 Use of unpaid care and of care services

We conducted multinomial logit regression analyses of CFASII to examine proportions of older people in each subgroup by age, gender, education, and severity of dementia who received no care, unpaid care only, formal community care only, both unpaid and formal community care, or care home services. We included education as an explanatory variable because the receipt of unpaid care and formal care varies with socio‐economic group with which education is closely associated. We used the fitted values from the regression model as the estimated proportions of each subgroup of the older population who received no care, unpaid care only, etc. We then applied these estimated proportions to the numbers of older people in each subgroup to estimate total numbers of older people nationally receiving unpaid care, formal community care, and care home services. We scaled the resulting national estimates for formal community care and care home services (but not unpaid care) to externally derived total numbers of older service users in England.^21^, ^24^ On the basis of CFASII, we assumed that 70% of older care home residents and 25% of older users of community care services in England have dementia.

### 2.5 Costing health and social care

We applied annual costs calculated from the MODEM cohort to our estimates of numbers of people with dementia using health, social, and unpaid care to calculate annual total costs in the older population with dementia. All health care costs are assumed to be met entirely by the NHS. Social care costs are divided between costs met by local authorities and those met by service users on an assumption that service users with dementia are divided between publicly and privately funded users in line with the breakdown for *all* older care service users in England.^24^

The MODEM cohort found considerable differences in service receipt between first and second interviews, except for those living in care homes. Since most members of the community sample were recruited from memory services, they are likely to have received a dementia diagnosis not long before their first interview. This may explain why they received more secondary health care but less formal social care at first interview than at second interview (12 months later). Therefore, we used service use data from the first interviews for the first year of care (incidence numbers) and data from the second interviews for second and subsequent years of care.

### 2.6 Costing unpaid care

Consistent with previous studies,^3^, ^10^, ^25^ we combined replacement cost and opportunity cost approaches to cost unpaid care, using data from the MODEM cohort on the proportion of carer time spent on: activities of daily living (ADL) tasks, instrumental activities of daily living (IADL) tasks, and supervision. These were estimated separately by the severity of dementia of care recipients and summed to estimate proportions of time spent on ADL tasks, IADL tasks, and supervision. At baseline, unpaid carers spent 10% of their caring time on assisting with ADLs, 19% assisting with IADLs, and 72% on supervision.

Total time spent on ADL tasks was calculated by multiplying the total time spent caring by the unpaid carer (from CSRI) by the proportion of time spent on ADL tasks (from BADLS). This was valued at £18 per hour, the replacement cost of an hour of formal home care.^20^ The remaining proportion of time spent caring was valued using an opportunity cost approach, assuming that carers who were not retired were forgoing employment to provide care. For each carer, this approach applied a value for unpaid care equal to the average wage for an individual with the same age, gender, and occupation (or the same age and gender if they did not report a specific occupation). For retired carers, the opportunity cost applied was the 2016 National Living Wage (£7.20 per hour) deflated to 2015 prices.^26^

The survey additionally asked about time provided by *other* carers. This time was also costed to reflect total unpaid care time received by the care recipient. Time contributed by other carers was allocated between ADL tasks, IADL tasks, and supervision based on the distribution of time reported by unpaid carer respondents. Replacement costs were applied to ADL time and the opportunity cost to IADL and supervision time. As data were not collected on the age, gender or occupation of other unpaid carers, the National Minimum Wage was taken as the opportunity cost for other unpaid carers.

### 2.7 One‐off and other costs

We included three types of one‐off costs, related to end‐of‐life care, diagnosis, and social care assessment. Since these are not included in the MODEM interviews, they are not included in our estimates for first year or subsequent year costs. End‐of‐life hospital cost for dementia has been estimated as 37.3% higher than for all conditions excluding cancer.^27^, ^28^ We inflated the estimate from these studies to 2015 prices to estimate end‐of‐life health care cost for dementia (£6415 per person). Median survival time of older people from onset of dementia is about 5 years.^29^ We therefore used incidence numbers in year 2010, estimated using CFASII data,^15^ as an estimate of numbers receiving end‐of life care in year 2015, and attached the estimated cost (£6415) to get the annualised total cost of end‐of‐life health care. Following previous work,^3^ diagnosis costs were added to health care costs, assessment costs were added to social care costs, and other costs (police, advocacy, and research) are reported as a separate category.

Our estimate of total annual costs comprises the sum of incidence numbers in 2015 multiplied by cost per person derived from the MODEM cohort first year interview, prevalence numbers minus incidence numbers in 2015 multiplied by cost per person derived from the MODEM cohort second year interview, and the one‐off costs and other costs.

## 3 RESULTS

### 3.1 Prevalence of dementia and service use

We estimate that 688 300 people had dementia in England in 2015, among whom 4.3% (37 500 people) have young‐onset dementia, defined as developing dementia before age 65 (Table 1). The estimated proportion of dementia is 6.7% among older people 65 and over, consistent with findings from other studies.^23^ The estimated proportions by severity in the older population are 16.8% mild (109 500 people), 36.6% moderate (237 900), and 46.6% severe (303 400). Among the 688 300 people with dementia, 564 100 receive health care, social care, or unpaid care, and 124 200 people receive neither social nor unpaid care. Ninety‐four percent of care recipients (530 900) are aged 65 or over, and 6% of the care recipients (33 200) have young‐onset dementia.

**Table 1:** Incidence and prevalence numbers by age bands and severity of dementia (thousand persons)

	Age Band					
	35‐64	65‐74	75‐84	85+	65+	All 35+
Population	20 968	5 282	3 131	1 297	9 710	30 679
Incidence rate (per thousand persons)	NA	6.0	23.5	49.3	18.9	NA
Incidence number	NA	31	68	50	171	NA
Prevalence rate, %	0.18	2.40	7.59	21.98	6.69	2.24
Prevalence number	38	127	238	285	650	688
Mild dementia (prevalence), %	0.10	0.43	1.44	3.23	1.13	0.43
Moderate dementia (prevalence), %	0.06	0.98	3.05	6.93	2.44	0.82
Severe dementia (prevalence), %	0.01	0.99	3.10	11.82	3.12	1.00

Sources: Population (ONS population estimates, 2015; see Office for National Statistics^11^); incidence rates for older people (CFASII for older people; see Matthews et al^15^); prevalence rates for older people (CFASII analysis); prevalence rates for early onset (Expert consensus); prevalence rates by severity for older people (CFASII analysis); prevalence rates by severity for early onset (assumed to be the same as older people with dementia aged 65‐69).

Around 251 000 older people with dementia live in care homes, of whom 80% (201 000) have severe dementia, while around 400 000 are community‐dwelling (Table 2); 258,000 older people in the community with dementia receive unpaid care, this being 40% of the overall older population with dementia and 65% of community‐dwelling older people with dementia. Around 90 000 older people with dementia receive formal social care in the community, which is 13% of the entire older population with dementia and 22% of community‐dwelling older people with dementia. Some people receive both unpaid and formal care.

**Table 2:** Estimated number of older people and younger adults with dementia receiving long‐term care by severity of dementia and care settings in England, 2015 (thousand persons)

	Mild Dementia		Moderate Dementia		Severe Dementia	Total
	No Dependency	With Care Needs	No Dependency	With Care Needs		
Older people						
Community care						
No care	19.8	17.3	31.3	43.0	8.6	119.9
Unpaid care only	2.0	26.1	11.2	93.1	60.8	193.3
Social care only	0.0	7.6	0.0	9.0	4.9	21.4
Both	0.0	7.7	0.0	29.6	28.1	65.3
Residential care	0.0	29.1	0.0	20.7	201.0	250.8
Total (older people)	21.8	87.7	42.5	195.4	303.4	650.8

	Mild Dementia		Moderate Dementia		Severe Dementia	Total
Younger adults						
Community care						
No care	3.8		0.5		0.0	4.3
Unpaid care only	3.6		0.7		0.0	4.3
Social care only	5.4		1.0		0.2	6.6
Both	7.8		2.6		0.3	10.7
Residential care	0.7		8.7		2.1	11.6
Total (younger adults)	21.5		13.4		2.6	37.5

Sources: Calculated from the MODEM cost‐of‐illness models.

### 3.2 Average annual costs per person with dementia

Average costs of mild, moderate, and severe dementia are £24 400, £27 450, and £46 050, respectively, per person per year. These are derived by dividing total costs by total prevalence numbers for each severity category. Table 3 shows the estimated average annual per‐person costs for health, social, and unpaid care in the older population. The MODEM cohort sample size was sufficient to break down the mild dementia group between those *with* care needs (low, medium, and high dependency) and those *without* care needs (independent), but was insufficient to conduct a similar breakdown for those with moderate dementia. Those with severe dementia all have care needs.

**Table 3:** Average costs of care for older people and younger adults with dementia in England, 2015 (£ per person per year, in 2015 prices)

	People Living in the Community				Care Home Residents
	Mild Dementia		Moderate Dementia	Severe Dementia	All with Dementia
	No Dependency	With Care Needs			
Older people					
First year					
Primary health care	400	550	400	450	225
Secondary health care	2625	2150	2775	4350	4575
Social care	0	1750	2600	4150	36 350
Unpaid care	6250	18 400	19 425	25 500	3450
First year total	9275	22 850	25 200	34 450	44 575
Second year					
Primary health care	475	525	425	350	300
Secondary health care	675	1850	1175	1575	4250
Social care	0	5350	10 350	13 875	34 800
Unpaid care	9650	13 975	20 775	26 700	3275
Second year total	10 775	21 700	32 725	42 500	42 625

	Mild Dementia		Moderate Dementia	Severe Dementia	All with Dementia
Younger adults					
Health care	2860		2777	11 636	9111
Social care	2572		7155	10 013	25 161
Unpaid care	19 714		31 966	34 247	2662
Total	25 146		41 898	55 896	36 934

Source: Calculated from the MODEM cohort data and the PSSRU Unit Costs of Health and Social Care.

For older people with dementia living in the community, estimated average annual costs of secondary health care are higher in the first year after diagnosis of dementia than in subsequent years (eg, £2775 vs £1175 for moderate dementia). Conversely, average annual costs for social care are much lower in the first year than in subsequent years (eg, £2600 vs £10 350 for moderate dementia). Average costs of unpaid care similarly rise between the first and second years, except for those with mild dementia and care needs, whose costs fall from £18 400 to £13 975. Mean total costs increase between the first and second year for all subgroups living in the community, except for people with mild dementia and care needs for which they remain similar. There is similarly a minimal difference in average overall costs between the 2 years for care home residents.

### 3.3 Total annual costs of dementia

Total annualised cost for people with dementia in England is £24.2 billion at 2015 prices (Table 4), of which 95% (£23.0 billion) is for older people, and the rest (£1.2 billion) is attributable to people with young‐onset dementia. Health care costs amount to £3.8 billion, including £0.9 billion attributable to end‐of‐life care. Health care costs are almost evenly split between people living in the community and in care homes. Total health care costs for people with mild, moderate, and severe dementia are £0.7 billion, £1.0 billion, and £2.1 billion, respectively.

**Table 4:** Total annualised costs of dementia for older people and younger adults combined in England, 2015 (£million, in 2015 prices)

	Mild Dementia	Moderate Dementia	Severe Dementia	Total
Community care				
Health care	480	710	610	1800
Social care	160	450	420	1030
Unpaid care	1250	4390	3910	9550
Other costs	19	53	24	96
Total	1910	5600	4970	12 480
Residential care				
Health care	180	270	1500	1950
Social care	1030	950	7130	9120
Unpaid care	60	70	450	580
Other costs	12	6	47	64
Total	1290	1300	9120	11 710
Total				
Health care	660	980	2110	3750
Social care	1190	1410	7550	10 150
Unpaid care	1320	4460	4360	10 130
Other costs	30	58	71	160
Total	3200	6900	14 090	24 190

Source: Calculated from the MODEM macrosimulation model.

Total social care costs are £10.2 billion, with £1.2 billion, £1.4 billion, and £7.6 billion attributable to mild, moderate, and severe dementia, respectively. Social care costs are heavily concentrated on people living in care homes (£9.1 billion). These costs of residential care relate mainly to people with severe dementia (£7.1 billion) since there are relatively few people with mild and moderate dementia in care homes. Social care costs for people living in the community are considerably smaller than residential care costs, being £1.0 billion in total, and £0.2 billion, £0.4 billion and £0.4 billion for people with mild, moderate, and severe dementia, respectively. We estimate that £6.2 billion (60.6%) of the total social care costs are met by users themselves and their families, with £4.0 billion (39.4%) funded by government.

Unpaid care is estimated to cost £10.1 billion, comprising £1.3 billion for people with mild dementia, £4.5 billion for moderate dementia, and £4.4 billion for severe dementia. Unpaid care costs are heavily concentrated on people living in the community (£9.6 billion) and much smaller for people living in care homes (£0.6 billion).

## 4 DISCUSSION

We report new figures for the economic impact of dementia in England using the best quality estimates of prevalence and new, detailed, and up‐to‐date measures of cost. In 2015, there were an estimated 688 300 people with dementia across England, with a total annual cost of £24.2 billion. Fifty‐sevenpercent of the total is attributable to health and social care service utilisation, with social care costs almost three times larger than health care costs. Unpaid care costs account for 42% of the total. Costs of severe dementia are £14.1 billion per year, twice the costs of moderate dementia (£6.9 billion) and 4.4 times larger than the costs of mild dementia (£3.2 billion). These are the *total* costs associated with supporting people with dementia, rather than the marginal extra costs “caused” by dementia. Costs of care relating to comorbidities are often higher for people with dementia than for people with other conditions, but the complication of disentangling what is and what is not a “dementia cost,” if such a distinction is indeed meaningful, is more a conceptual than an empirical issue.

### 4.1 Previous studies

Prince et al^3^ reported the total annual costs of dementia in the United Kingdom to be £26.3 billion, including £22.1 billion in England. Our new estimate for England is around £2 billion higher. The reasons for this difference are threefold. First, we used data from CFASII, which found a higher proportion of older people with severe dementia than previously estimated. The prevalence rates of dementia calculated using CFASII were not available at the time of that previous work, but are now being used by NHS England as the official figures.^1^, ^30^ Second, we used data from CFASII instead of randomised controlled trial samples to estimate the number of care recipients with dementia. Compared with the trials data (N = 1462), CFASII (N = 7764) has a much larger sample size and does not impose restrictions on the characteristics of older people with dementia in the recruitment process. Third, we used MODEM cohort data instead of the trials data to estimate the unit costs of health, social, and unpaid care. Not only are MODEM cohort data more recent, which is especially important given recent cutbacks in (for example) social care spending, but they also relate to a general sample of people receiving services rather than a more specific (and almost certainly less representative) group consenting to participate in trials. We have thus addressed some of the limitations of the two previous UK estimates by using data from surveys, which are more recent, have larger samples, and are more representative of people with dementia than data sources used in the earlier studies.

Consistent with similar studies in other countries,^31^ we find that unpaid care accounts for a substantial proportion of the total cost of dementia. It is projected that both the number and proportion of the older population, especially those aged 85 and over (among whom prevalence rates of dementia are highest), will continue to increase rapidly in England.^11^ The demand for and costs of unpaid care for people with dementia can be expected to grow substantially in the coming decades.

### 4.2 Strength and limitations

Drawing on newly available data from multiple sources including 2015 ONS population data, NHS Digital data on receipt of publicly funded social care, the National Living Wage, CFASII, and MODEM, our study presents comprehensive, up‐to‐date evidence on a range of costs including those associated with end‐of‐life care and young‐onset dementia. With more detailed data, we are able to explicitly model unpaid carers' caregiving activities and adopt better theory‐based approaches to calculate unpaid care costs.^32^ Building on, but moving beyond previous work, our refined modelling approaches ensure additional rigour and robustness. In particular, the use of separate data on use of services in the first year and in the second year after diagnosis is a distinct advance on previous work.

Three limitations of the study should be noted. First, the estimated costs of unpaid care are sensitive to the methodology for estimating the opportunity costs of unpaid care; but this limitation is inevitable when studying the costs of a condition for which much of the care is provided by unpaid carers.^32^ Second, unpaid carers themselves may have care needs and use health and social care services, but the costs relating to these services were not included as we could not find suitable data to disentangle which costs could be attributed to caregiving activities. Third, drawing on data from multiple sources also means that we have to deal with a “patchwork” of data. In particular, some data used in the analyses involve individuals from specific regions of England and thus may not be representative of the entire population of people with dementia. This limitation is, however, shared with most research.

### 4.3 Policy implications

People with dementia receive combined support from health care and social care professionals and unpaid carers. Hence, coordination, synergy, and mutual support between sectors should be encouraged to better serve the needs of people with dementia and tackle the associated social and economic challenges. Given the substantial contribution of unpaid carers and the scale of unpaid care costs, support is essential for those carers, in order to promote their health and well‐being and enable them (if they wish) to combine caring with employment or other activities. This support could include increased information and advice services, increased resources for respite care, or increased cash payments to carers.

The economic impact of dementia is not evenly shared between the health and social care systems, but weighs heavily on the already underfunded social care sector. The reduction in central government funding for local authorities in recent years has impacted on resources for social care leading to a decline in the numbers of older people receiving publicly funded community‐based and residential care. Unlike health care that is free of charge at the point of use in England, the entitlement to publicly funded social care depends upon service users' income and assets, and eligibility criteria for publicly funded care vary considerably across England due to the discretionary power of local authorities.^33^ Around 70% of care home residents have dementia,^23^ and a substantial proportion of the social care costs is met by people with dementia or their families. These findings further highlight the importance of addressing the challenges of social care financing. In particular, social care should be financed fairly and efficiently to make sure that high‐quality care services can be delivered in a timely fashion to those people who need them.

## DATA AVAILABILITY

The modelling uses a range of data sets, as explained above and in Figure 1. The ONS data are available at www.ons.gov.uk. The NHS Digital data are available at www.digital.nhs.uk/data‐and‐information/areas‐of‐interest/social‐care. The CFASII data are available by application to the CFAS research team as explained at www.cfas.ac.uk. The MODEM study data will be deposited with the UK Data Archive.

## CONFLICT OF INTEREST

None declared.

## Supporting information

Data S1. The costs of dementia in England: Supplementary table

## References

[1] NHS England . Dementia. Leeds: NHS England; 2018 https://www.england.nhs.uk/mental‐health/dementia/. Accessed on 15 August 2018.

[2] Kingston A , Robinson L , Booth H , Knapp M , Jagger C . MODEM project. Projections of multi‐morbidity in the older population in England to 2035. Age Ageing. 2018;47(3):374‐380. 10.1093/ageing/afx201 29370339 PMC5920286

[3] Prince M , Knapp M , Guerchet M , et al. Dementia UK: Update. London: Alzheimer's Society; 2014 https://www.alzheimers.org.uk/sites/default/files/migrate/downloads/dementia_uk_update.pdf. Accessed on 15 August 2018.

[4] Byford S , Torgerson DJ , Raftery J . Cost of illness studies. BMJ. 2000;320(7245):1335 10.1136/bmj.320.7245.1335. 10807635 PMC1127320

[5] L‐JE K , Pai M‐C , Shih P‐Y . Economic impact of dementia by disease severity: exploring the relationship between stage of dementia and costs care in Taiwan. PLoS ONE. 2016;11(2):e0148779 10.1371/journal.pone.0148779 26859891 PMC4747483

[6] Hojman DA , Duarte F , Ruiz‐Tagle J , Budnich M , Delgado C , Slachevsky A . The cost of dementia in an unequal country: the case of Chile. PLoS ONE. 2017;12(3):e0172204 10.1371/journal.pone.0172204 28267795 PMC5340351

[7] Prince M , Wimo A , Guerchet M , Ali G‐C , Wu Y‐T , Prina M . The Global Impact of Dementia: An Analysis of Prevalence Incidence, Cost and Trends. London: Alzheimer's Disease International; 2015 https://www.alz.co.uk/research/world‐report‐2015. Accessed on 15 August 2018.

[8] Romeo R , Knapp M , Salverda S , Orrell M , Fossey J , Ballard C . The cost of care homes for people with dementia in England: a modelling approach. Int J Geriatr Psychiatry. 2017;32(12):1466‐1475. 10.1002/gps.4637 27911013

[9] Quentin W , Riedel‐Heller SG , Luppa M , Rudolph A , Konig H‐M . Cost‐of‐illness studies of dementia: a systematic review of focusing on stage dependency of costs. Acta Psychiatr Scand. 2010;121(4):243‐259. 10.1111/j.1600-0447.2009.01461.x 19694634

[10] Knapp M , Prince M , Albanese E , et al. Dementia UK. London: Alzheimer's Society; 2007.

[11] Office for National Statistics . 2014‐based National Population Projections, England. London: Office for National Statistics; 2015 Available at: https://www.ons.gov.uk/peoplepopulationandcommunity/populationandmigration/populationprojections/datasets/tablea24principalprojectionenglandpopulationinagegroups Accessed on 15 August 2015.

[12] Kingston A , Jagger C. *Population Ageing and Care Simulation Model (PACSim): Baseline Dataset and Model Construction (version: 241017)* 2017 http://docs.wixstatic.com/ugd/e1a359_b0ae66e642ac4263b67c629c9eb22543.pdf?index=true. Accessed on 16 August 2018.

[13] Kingston A , Comas‐Herrera A , Jagger C . Forecasting the care needs of the older population in England over the next 20 years: estimates from the Population Ageing and Care Simulation (PACSim) modelling study. Lancet Public Health. 2018;3(9):August. 10.1016/S2468-2667(18)30118-X):e447‐e455. 30174210 PMC6123499

[14] Isaacs B , Neville Y . The needs of old people: the ‘interval’ as a method of measurement. J Epidemiol Community Health. 1976;30(2):79‐85. 10.1136/jech.30.2.79 PMC478944 953380

[15] Matthews FE , Stephan BCM , Robinson L , et al. A Two decade dementia incidence comparison from the Cognitive Function and Ageing Studies I and II. Nat Commun. 2016;7(1):11398). 10.1038/ncomms11398. 27092707 PMC4838896

[16] Comas‐Herrera A , Knapp M , Wittenberg R , et al. MODEM: a comprehensive approach to modelling outcome an costs impacts of interventions for dementia. Protocol Paper. BMC Health Serv Res. 2017;17(1):1‐8. 10.1186/s12913-016-1945-x 28077155 PMC5225619

[17] Beecham J , Knapp M . Costing psychiatric interventions In: Thornicroft G, ed. Measuring Mental Health Needs. London: Gaskell; 2001.

[18] Bucks RS , Ashworth DL , Wilcock GK , Siegfried K . Assessment of activities of daily living in dementia: development of the Bristol Activities of Daily Living scale. Age Ageing. 1996;25(2):113‐120. 10.1093/ageing/25.2.113 8670538

[19] Wimo A , Gustavsson A , Jonsson L , Winblad B , Hsu M‐A , Gannon B . Application of resource utilization in dementia (RUD) instrument in a global setting. Alzheimers Dement. 2013;9(4):429‐435.e17. 10.1016/j.jalz.2012.06.008 23142433

[20] Curtis L , Burns A . *Unit Costs of Health and Social Care 2017* University of Kent: Personal Social Services Research Unit; 2017 10.22024/UniKent/01.02/65559.

[21] NHS Digital . Community Care Statistics, Social Services Activity, England, 2015‐16 Report. Leeds: NHS Digital; 2016 http://webarchive.nationalarchives.gov.uk/20180328140255/http://digital.nhs.uk/catalogue/PUB21934. Accessed on 15 August 2018.

[22] Copeland JRM , Dewey ME , Griffiths‐Jones HM . A computerized psychiatric diagnostic system and case nomenclature for elderly subjects: GMS and AGECAT. Psychol Med. 1986;16(01):89‐99. 10.1017/S0033291700057779. Published 3515380

[23] Matthews FE , Arthur A , Barnes LE , et al. A two‐decade comparison of prevalence of dementia in individuals aged 65 years and older from three geographical areas of England: results of the cognitive function and ageing study I and II. The Lancet. 2013;382(9902):1405‐1412. 10.1016/S0140-6736(13)61570-6. PMC3906607 23871492

[24] Wittenberg R , Hu B , Hancock R . Projections of Demand and Expenditure on Adult Social Care 2015 to 2040. *PSSRU Discussion Papers, DP2944* London: Personal Social Services Research Unit; 2018 http://eprints.lse.ac.uk/5136/. Accessed on 15 August 2018.

[25] Wimo A , von Strauss E , Nordberg G , Sassi F , Johansson L . Time spent on informal and formal care giving for persons with dementia in Sweden. Health Policy. 2002;61(3):255‐268. 10.1016/S0168-8510(02)00010-6 12098519

[26] UK Government . National Minimum Wage and National Living Wage Rates. London: UK Government; 2018 https://www.gov.uk/national‐minimum‐wage‐rates. Accessed on 15 August 2018.

[27] Georghiou T , Bardsley M . Exploring the Cost of Care at the End of Life. London: Nuffield Trust; 2014 https://www.nuffieldtrust.org.uk/files/2017‐01/end‐of‐life‐care‐web‐final.pdf. Accessed on 15 August 2018.

[28] Georghiou T , Davies S , Davies A , Bardsley M . Understanding Patterns of Health and Social Care at the End of Life. London: Nuffieldtrust; 2012 https://www.nuffieldtrust.org.uk/files/2017‐01/understanding‐patterns‐health‐social‐care‐end‐of‐life‐full‐web‐final.pdf. Accessed on 15 August 2018.

[29] Xie J , Brayne C , Matthews FE , MRC CFAS collaborators . Survival times in people with dementia: analysis from population based cohort study with 14 year follow‐up. BMJ. 2008;336(7638):258 10.1136/bmj.39433.616678.25‐262. 18187696 PMC2223023

[30] NHS Digital . Recorded dementia diagnoses—June 2018. Leeds: NHS Digital; 2018 https://digital.nhs.uk/data‐and‐information/publications/statistical/recorded‐dementia‐diagnoses/june‐2018#key‐facts. Accessed on 15 August 2018.

[31] Wimo A , Gauthier S , Prince M . Global Estimates of Informal Care. London: Alzheimer's Disease International; 2018 https://www.alz.co.uk/adi/pdf/global‐estimates‐of‐informal‐care.pdf. Accessed on 15 August 2018.

[32] Rehill A , Comas‐Herrera A , Farina N , King D , Knapp M , Lorenz K , Peña Longobardo LM (in preparation) The impact of different question formulations on estimates of unpaid care provision.

[33] Comas‐Herrera A , Wittenberg R , Pickard L . The long road to universalism? Recent developments in the financing of long‐term care in England. Soc Policy Adm. 2010;44(4):375‐391. 10.1111/j.1467-9515.2010.00719.x

