# Supplementary material for: The costs of dementia in England
Source: Int J Geriatr Psychiatry. 2019 Apr 24;34(7):1095–103. doi: 10.1002/gps.5113 (PMC6618309; doi:10.1002/gps.5113)
Supplement: Supplementary file 1 — Data S1. The costs of dementia in England: Supplementary table [file GPS-34-1095-s001.docx]

**The Costs of Dementia in England: Supplementary Table**

Table A1: Use of services by the MODEM study cohort (at first interview) and unit costs of services, 2015/2016 prices

The MODEM cohort sample baseline (first) interviews took place between June 2015 and October 2016. 307 individuals living with dementia and their primary carer were interviewed.

| Service | Number of users | Average frequency of use among users | Unit cost | Source |
| --- | --- | --- | --- | --- |
| **Accommodation** |  |  |  |  |
| Residential care home – LA | 1 | 365 days in last year | £782 per week | PSSRU Unit Costs, 2016. Page 204. Average of min and max cost of a shared room. |
| Residential care home - Private | 23 | 242 days in last year | £518 per week | PSSRU Unit Costs, 2016. Page 204. Average of min and max cost of a shared room. |
| Nursing home – LA or NHS | 5 | 312 days in last year | £701 per week | PSSRU Unit Costs, 2016. Page 204. Average of min and max cost of a shared room. |
| Nursing home - Private | 29 | 284 days in last year | £701 per week | PSSRU Unit Costs, 2016. Page 204. Average of min and max cost of a shared room. NB. Same costs for-profit and not-for-profit listed |
| Care home/nursing home short stays | 21 | 14 days in last 3 months | £151.86 per night | Weekly establishment cost (only) for LA (assumed) residential care divided by 7. |
| **Hospital** |  |  |  |  |
| Inpatient stay - Psychiatry | 2 | 41 days in the last 3 months | £429 per day | NHS Reference Costs, 2015-16– Mental Health – Specialist Mental Health Services – Other admitted patient |

| Service | Number of users | Average frequency of use among users | Unit cost | Source |
| --- | --- | --- | --- | --- |
| Inpatient stay - General | 34 | 8 days in the last 3 months | £395 per day | NHS Reference Costs, 2015-16. Average of National Average Unit Cost across specialties for elective inpatient excess bed days |
| Day case | 6 | 1 day in the last 3 months | £733.31 per case | <https://www.gov.uk/government/uploads/system/uploads/attachment_data/file/577084/National_schedule_of_reference_costs_-_main_schedule.xlsx> - NHS reference cost 2015-16. |
| Memory clinic | 94 | 2 appointments in the last 3 months | £428 per hour | PSSRU Unit Costs, 2016. Page 31 |
| Hospital outpatient | 105 | 2 appointments in the last 3 months | £116.92 per visit | <https://www.gov.uk/government/uploads/system/uploads/attachment_data/file/577084/National_schedule_of_reference_costs_-_main_schedule.xlsx> - NHS reference cost 2015-16. |
| A&E | 44 | 1 visit in the last 3 months | £146.86 per visit | NHS Reference Costs 2015-16. Total Outpatient Attendances |

| Service | Number of users | Average frequency of use among users | Unit cost | Source |
| --- | --- | --- | --- | --- |
| **Community health and social care** |  |  |  |  |
| Paramedic | 49 | 1 use in the last 3 months | £96.25 per use | NHS Reference Costs 2015-16. Index. Unit cost for ambulance |
| Psychiatrists | 19 | 1 visit in the last 3 months | £106 per visit | PSSRU Unit Costs, 2016. Page 191. Consultant: psychiatric |
| General Practitioner | 212 | 2 visits (inclusive of phone consultations) in the last 3 months | £31 per home visit; £31 per clinic visit lasting 9.2min; £14.60 per phone consultation | PSSRU Unit Costs, 2016. Page 145,147. Home visit costs taken as same as clinic visit as not sure what basis for premium to use (travel time? Visit duration?). GP phone consultation unit costs from GP-led telephone triage. |
| NHS 111 | 28 | 2 contacts in the last 3 months | £8.50 per contact (contracted cost) | <http://www.bbc.co.uk/news/health-22370621> |
| Community psychiatric nurse | 36 | 2 visits in the last 3 months | £67 per hour (face-to-face time) | PSSRU Unit Costs, 2015. Page 170. |
| District nurse | 22 | 3 visits in the last 3 months | £44 per hour | PSSRU Unit Costs, 2016. Page 142. Band 6. As above, home visit costs taken as same as clinic visit. |

| Service | Number of users | Average frequency of use among users | Unit cost | Source |
| --- | --- | --- | --- | --- |
| Practice nurse | 77 | 2 visits in the last 3 months | £36 per hour  £6.10 per telephone contact (or £0.93 per telephone minute) | PSSRU Unit Costs, 2016. Page 143,147. Home visit costs taken as same as clinic visit as not sure what basis for premium to use (travel time? Visit duration?). Practice nurse phone consultation unit costs from nurse-led telephone triage. |
| Dentist | 102 | 1 visit in the last 3 months | £122 per visit | PSSRU Unit Costs, 2016. Page 148. Unit cost per hour of patient contact. |
| Home care | 67 | 23 hours per week | £18 per hour | PSSRU Unit Costs, 2016. Page 160. Assumes independent sector home care rate. |
| Day care | 58 | 2 sessions per week | £61 per attendance | PSSRU Unit Costs, 2016. Page 28. Assumes each attendance is for 3.5 hours in a local authority day care facility |
| Social club | 33 | 1 session per week | £5.62 per session | Romeo et al., 2013. Cost-effectiveness analyses for mirtazapine and sertraline in dementia: randomised controlled trial. Br J Psych; 202(2):121-128. Uprated using HCHS inflator. |

REFERENCES

Curtis L, Burns A. (2016). Costs of Health and Social Care 2016. Personal Social Services Research Unit: Canterbury.

Department of Health (2016) NHS reference costs 2015-2016. <https://www.gov.uk/government/publications/nhs-reference-costs-2015-to-2016>. Accessed: 22^nd^ January 2019.

Romeo et al., 2013. Cost-effectiveness analyses for mirtazapine and sertraline in dementia: randomised controlled trial. Br J Psych; 202(2):121-128.
